# Closing the academy–Business gap by building intellectual capital in professional formation

**DOI:** 10.3389/fsoc.2023.969285

**Published:** 2023-05-04

**Authors:** Adela M. Vélez-Rolón, Alejandra Pulido López, Manuel Méndez-Pinzón, Diego Neira-Bermudez

**Affiliations:** ^1^School of Management, Colegio de Estudios Superiores de Administración, Bogotá, Colombia; ^2^School of Management and Business, Universidad del Rosario, Bogotá, Colombia; ^3^School of Management and Business, Politécnico Grancolombiano, Bogotá, Colombia; ^4^School of International Business, Fundación Universitaria del Área Andina, Bogotá, Colombia

**Keywords:** professional formation, intellectual capital, SDG 8, knowledge management, decent work and economic growth

## Abstract

**Introduction:**

The rapid advances in technology, market pressures, globalization, and, recently, the COVID-19 pandemic show the need to find educational models that respond to these realities while improving the employability levels of young people and promoting economic growth. This research analyzes how the professional formation model, where two learning spaces, the academy, and the company, are combined, promotes the closing of gaps and economic growth, through the development of intellectual capital that arises from this relationship, in an emerging economy such as Colombia.

**Methods:**

The methodology used corresponds to a qualitative approach, where the vision of the actors involved in the training process at the undergraduate level in Colombia is analyzed through semi-structured interviews, focus groups, and documentary analysis.

**Results and discussion:**

The results show five major elements to consider for the formation of the intellectual capital required for the success of the relationship and the reduction in the gaps between academia and business: decision-making mechanisms, inter-organizational coordination, knowledge.

## Introduction

Technological progress, globalization, the market, and a volatile, uncertain, complex, and ambiguous environment (VUCA) have driven knowledge as the key factor that promotes competitiveness creation, growth, and wealth of the countries, contributing to facing complex problems that affect humanity and that are highlighted in the 2030 Agenda. In this context, intellectual capital must be understood as the force that allows the resolution of these problems.

One of the major issues is youth unemployment. Young people face difficulties in labor markets, precariousness and predominance of low-skilled jobs, low work experience, occupational turnover and informality, income instability, lack of legal protection, and limited training and career opportunities, which generate occupational instability and do not allow them to acquire the qualifications, experience, and skills necessary for future job stability (International Labour Office, 2020; Organización Internacional del Trabajo, 2021). In addition, they are three times more likely than adults to be unemployed (International Labour Office, 2020).

According to the International Labour Organization, young people in Latin America returned to employment possibilities more quickly than adults after the crisis generated by the COVID-19 pandemic, a phenomenon explained by youth informality in the labor market and the recovery of the activities with the highest youth employment; however, the youth unemployment rate in 2020 and 2021 was ~22% (Organización Internacional del Trabajo, 2021), which is higher than the global rate of 13.6% in 2020, becoming this a matter of concern in the region. By 2020, globally, one-fifth of young people were not gaining experience or income from work, nor were they improving their education levels or skills (International Labour Office, 2020).

Regardless of the sector of the economy, companies require employees with knowledge and skills in information technologies that support activities and technical functions in different areas (International Labour Office, 2020). Having a university education degree reduces the risk of job displacement due to process automation, given the skills acquired for problem-solving, which generates the challenge of ensuring quality academic programs with sufficient coverage and closing the gap between business and academy, bringing, consequently, the challenge that will lead to the development of solutions to the affairs that afflict the world nowadays.

Dual training is the model that has brought the business sector and academia closer, also known as Vocational Education and Training (VET) systems. In 2001, Colombia implemented this vocational training model through a cooperation agreement between the German government and the chambers of commerce, to seek competitiveness and improve the productivity of companies in the country, closing the gaps in training.

Studies analyze this type of training in different parts of the world, and some of these focused on transfer (Euler, 2013; Gonon, 2014; Hinrichs, 2014; Gessler, 2017b); in Latin America, it is described (Rojas, 2015; Molina, 2016; Espinoza Freire, 2020; Peguera Carré et al., 2021; Zapata et al., 2021), focusing on the experience since its implementation, analyzing the gaps and challenges for its understanding, limitations, and possibilities, and being this a potential field for research considering the contribution of this model to economic growth and decent work as it has been proposed worldwide (Vélez Rolón, 2019).

### Knowledge management in professional formation

Drucker (1993) suggests the relevance of understanding knowledge as the most valuable resource for economic growth, in the so-called knowledge society. In organizations, this knowledge must be created from the interaction of the tacit knowledge of each member and the explicit knowledge created in the organization (Nonaka and Takeuchi, 1995; Nonaka and Konno, 1998; Von Krogh et al., 2000). This generates value for the organization, taking advantage of the use of knowledge as an intangible asset, the intensive use of technology and processes efficiency, and the development of innovations (Hislop et al., 2018).

This occurs from two fundamental processes: first, knowledge management, assumed as the identification, capture, systematization, and use of organizational knowledge (Nonaka and Takeuchi, 1995; Firestone and McElroy, 2003; Senge and KIm, 2013), and second, the transfer of knowledge seen as the bidirectional process flow of knowledge; from the theory of communication, this flow requires three determinants: the willingness of the transfer or the receiver; the appropriate channels of transmission; and understanding the value and use of the knowledge transferred (Gupta and Govindarajan, 2000).

### Intellectual capital

Intellectual capital has been studied from the value creation and the competitive advantage of organization approaches. Edvinsson (1997) defines intellectual capital as the set of knowledge, experience, technology, customer relationships, and professional skills that an organization has; it is the knowledge assets that turn into value and create innovation (Sardo et al., 2018), from the creation and connection between expertise, experience, and competence inside and outside organizations (Do Rosario et al., 2008), and is conceived as a source of competitive advantage (Sharabati et al., 2010). This intellectual capital, understood as intangible assets, does not appear on the company balance sheets but generates more value for organizations than physical assets (Hashim et al., 2015). This generation of value and competitive advantage comes from the combination of the dimensions of intellectual capital: human capital, structural capital, and relational capital (Do Rosario et al., 2008; Kianto et al., 2017; Sardo et al., 2018; Li et al., 2021).

Human capital is considered the main source of competitive advantage (Do Rosario et al., 2008), as it is a source of renewal, creativity, and innovativeness (Sardo et al., 2018), and includes the knowledge, qualifications, skills (Zeghal and Maaloul, 2010), experience, commitment, and motivation (Kianto et al., 2017) of an organization's employees. It is the individual stock of knowledge that is further nurtured by their willingness, skills, and training (Li et al., 2021). This capital is not owned by the company since it leaves as soon as members of the organization leave (Zeghal and Maaloul, 2010; Kianto et al., 2017; McDowell et al., 2018).

On the contrary, structural capital is understood as the non-human assets of an organization, which are embodied and stored in information systems, databases, programs (Do Rosario et al., 2008), production processes, information technologies (Zeghal and Maaloul, 2010), information systems, work procedures, know-how (Edvinsson and Sullivan, 1996), innovations, business processes (Roos and Whitehill, 1998; Do Rosario et al., 2008), and organizational capabilities, culture, and intellectual property (Sardo et al., 2018). This capital is owned by the organization and remains in the organization when employees or members of the organization are no longer part of it.

Finally, relational capital, sometimes known as social capital (Subramaniam and Youndt, 2005; Kianto et al., 2017; Li et al., 2021) or customer capital (Bontis, 1998), includes relationships and knowledge with customers, suppliers, industry, partners, connections and relationships with authority (Do Rosario et al., 2008; Sharabati et al., 2010; Kianto et al., 2017; Li et al., 2021), and stakeholders and also includes brand loyalty, image, and reputation (Sardo et al., 2018).

### Professional formation

Tertiary education models have been changing and evolving in ways that allow them to respond to the needs of education and the growing demands of the market and the pressures of the complex problems of the twenty-first century. Hanna (1998) classifies these new models from the point of view of education providers, considering the abovementioned pressures, a classification that is still relevant and has given some relevance to models about contextual pressures. In this research, models that reflect the business-academy alliance are analyzed.

Etzkowitz and Leydesdorff (1995) encompassed this alliance from the triple helix model, which explains the relationship between government, industry, and universities for the production of knowledge and innovation; later, a fourth helix was included that sought to explain these relationships enclosed by social complexities (Carayannis and Rakhmatullin, 2014), arguing that the relationships between these actors must be understood within a system that allows the production and diffusion of knowledge (Lundvall, 2010). It is in this context of knowledge production, in which academy–business cooperation networks become a driver of economic growth while allowing universities to play a different role in generating value for society (De Fuentes and Dutrénit, 2012; Sam and van der Sijde, 2014; Bikse et al., 2016). Faced with this reality, this training model, developed in Germany, has taken great relevance to approach the academy–industry relations and to close the gap between academia and the productive sector.

The model is based on the training of young people in two alternate spaces: the academy and the company, implemented in different ways around the world (Eichhorst et al., 2015). Such variation in models is linked to the development of training itself, pressured by historical and cultural processes in each country, which determines the way how it should be adapted (Euler, 2013; Gessler and Howe, 2015). Some of the characteristic elements of dual training are as follows: (1) having two learning spaces, (2) job rotation during the professional internship, (3) the existence of a work plan created between teachers and company instructors, and (4) the existence of tutors in the classroom and the company.

High youth unemployment rates, coupled with the need to train competent human capital, have revived the debate on the relevance of implementing dual training models, bringing several challenges.

In this scenario, companies face obstacles when developing dual training programs. One major obstacle is financial, deepened by the crisis generated by the COVID-19 pandemic (OECD, 2021); the incorporation of technology, remote work, and flexibility to take breaks during work periods were some of the mechanisms used by companies that are expected to continue to counteract the effects of the crisis in the short and medium term and develop in this way training and VET. That is why higher education institutions are the ones that must take on the challenge of generating spaces and strategies that offer the possibility of practical learning through simulations and other information technologies, applied projects, the use of laboratories, and other experiential learning strategies (OECD, 2021). In this sense, and to ensure higher levels of employability of young people, integrated policies are required to promote research and development that foster new sectors and innovations leading to job creation and, on the contrary, to promote the updating of the educational offer taking into account market needs and trends that include personal and digital skills (International Labour Office, 2020).

In this context, the Organisation for Economic Co-operation and Development (2020) recommends actions for the construction of VET systems in future by countries. Among these, it proposes the interaction between employers and unions, planning for changes in the labor market, financial aid, availability of digital and distance education, promotion of digital badges, development of transversal competencies, incorporation of vulnerable groups, and teacher qualification (Organisation for Economic Co-operation and Development, 2020).

## Research questions

The study addresses the following research question: How can professional formation contribute to the development of intellectual capital and bridge the gap between academia and business in emerging economies? case Colombia.

## Data and methods

The research methodology chosen was the case study, which allows for the examination in depth of the dynamics and relationships of a phenomenon under study (Yin, 2009), allowing to establish relationships between cases (Hancock and Algozzine, 2011). For the selection of these cases, a non-probabilistic purposive sample was selected, which is characterized by allowing the researcher to define according to the need of the research. The case studies chosen for this research correspond to the five higher education institutions (HEI) that have had education programs with professional formation models in Latin America, part of the *Duale Hochschule Latinoamérica (DHLA)* (DHLA).

For each case study, the same strategy of individual analysis was established to continue with the cross-checking of data and the construction of relationships and overviews. The research presents a qualitative approach, in which different data sources and data collection techniques were used to validate the results, through a triangulation process, and the information was organized in a database analyzed through the ATLAS Ti 22 software, where the chain of evidence for each case was maintained (Yin, 2009).

The qualitative research techniques used were as follows: 16 semi-structured interviews with professors, internship supervisors, businessmen, and managers of the HEI; analysis of 43 official documents of the model corresponding to guidelines, institutional strategic plans, internship follow-up plans, web pages, and improvement plans made by students; additionally, 10 group interviews were conducted with students of the five HEI.

For the data collection, it was necessary to identify the actors such as professors, internship supervisors, entrepreneurs, and managers of the HEI and to develop profiles for each one, generating the codes for their respective identification in the matrixes where the results are compared, and the instrument and size of the sample were also codified for each HEI, as well as the main characteristics and general information of each institution.

The methodological process was carried out from the documentary analysis and data collection through interviews based on the selected theoretical variables. Subsequently, the individual and group interviews were transcribed, generating a list of categories and assigning them to each question to finish with the analysis, categorization, and development of the reports.

### Data analysis

**Figure 1:**
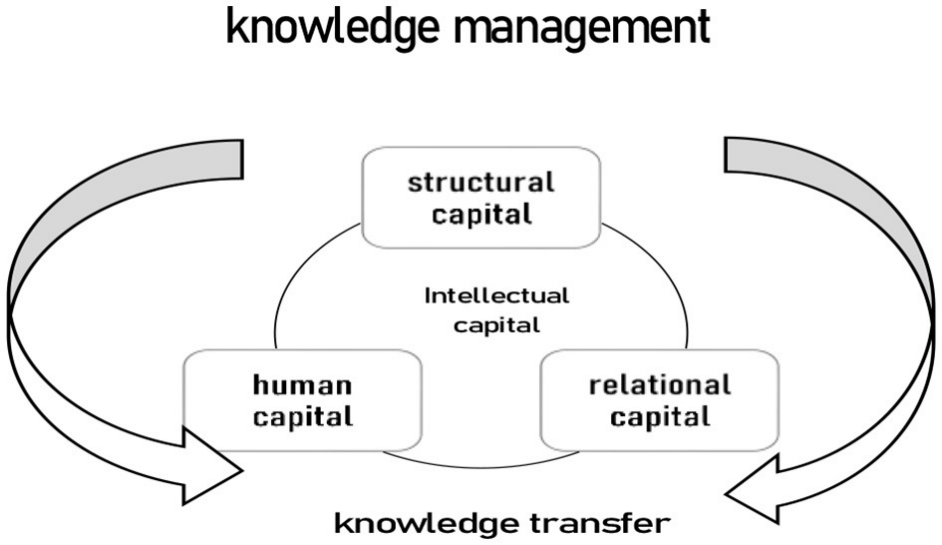
Knowledge management. Source: Author's elaboration.

In this process, the responses of the participants were categorized and codified, grouping them in an orderly manner, facilitating the comprehension of the categories identified theoretically (see Table 1); the variables used for the data analysis gave rise to the deductive categorization (see Figure 1), which allowed understanding the construction of intellectual capital.

**Table 1:** Theoretical constructs.

**Variable**	**Definición**	**Autores**
Knowledge management	This variable describes the activities that generate knowledge in the company, its relationship with innovation, and the knowledge cycle.	Nonaka and Takeuchi, 1995; Nonaka and Konno, 1998
Human capital	It refers to the knowledge, skills, qualifications, experience, commitment, training, willingness, motivation, and aptitudes of the members of an organization.	Zeghal and Maaloul, 2010; Kianto et al., 2017; McDowell et al., 2018; Li et al., 2021
Structural capital	It refers to the non-human assets of an organization: information systems, databases, software, production processes, information technologies, information systems, work procedures, know-how, innovations, business processes, organizational capabilities, culture, and intellectual property.	Edvinsson and Sullivan, 1996; Roos and Whitehill, 1998; Do Rosario et al., 2008; Zeghal and Maaloul, 2010; Sardo et al., 2018
Relational capital	This variable includes relationships and knowledge of customers, suppliers, industry, partners, connections, relationships with authority, stakeholders, brand loyalty, image, and reputation.	Sharabati et al., 2010; Kianto et al., 2017; Sardo et al., 2018
Knowledge transfer	The variable refers to the mechanisms that enable the flow of knowledge.	Gupta and Govindarajan, 2000

Source: Author's elaboration.

The information extracted from the documents was analyzed, and both individual and group interviews and observations were transcribed, categorizing and classifying each variable proposed in a database using the qualitative data analysis software ATLAS TI v22. For this analysis, lists were constructed with the categories to be assigned to each case, as defined by Hernández Sampieri et al. (2014), giving values that facilitate the identification of each item of data; this list of codes and categories was based on the literature, taking as a basis a documentary analysis of the theoretical foundations found for the construction of the instruments used.

#### Deductive encoding

This step began with the assignment of sections of interviews, individual and group, documents to each of the categories identified from the theoretical references of the research, called units of significance (US), which represent fragments of the interviews, focus groups, and documents analyzed that are associated with each theoretical category defined.

#### Inductive encoding

For this encoding, it was necessary to periodically review and add codes when necessary, and the 800 (US) were grouped into codes from the data, finding 37 codes that allow to understand the construction of intellectual capital in professional formation. This process concluded when the units of significance were repeated in each of the cases, and when they were assigned to the categories and finally to the codes.

#### Emerging categories

The analysis of the data results in the development of *five* metacategories that allow the recognition of the most important elements to consider for the development of intellectual capital in the professional formation model in Colombia, which at the same time explains how they favor economic growth when closing the *academy*–*business* gaps.

## Results

### Deductive codification

**Figure 2:**
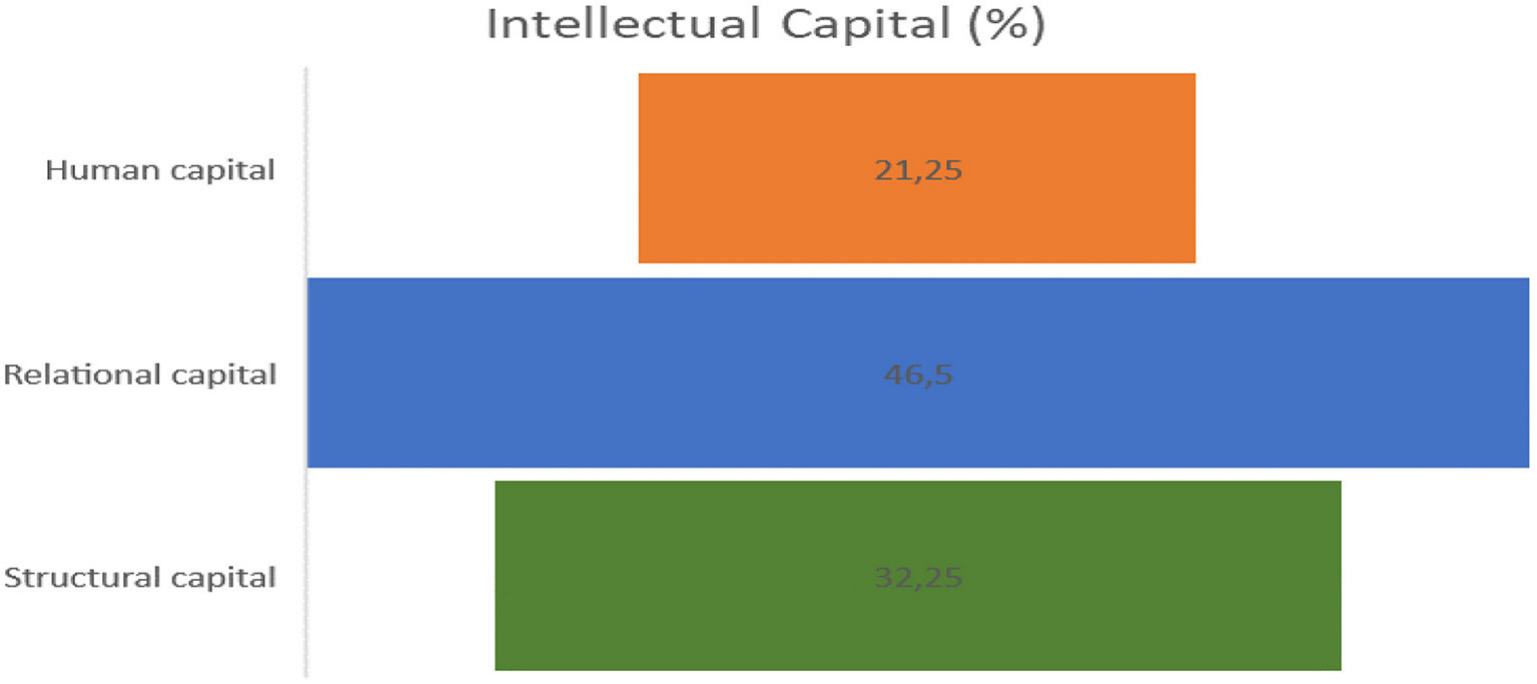
Intellectual capital. Source: Author's elaboration.

The deductive codification process was carried out by considering the variables proposed from the theory that allows the development of intellectual capital: relational capital (46.5%), human capital (21.25%), and structural capital (32.25%). A total of 800 units of meaning were found from the analysis of the different data collected (Figure 2).

### Inductive codification

Based on the contributions made by the participants and the analysis of the official documents, 37 indicative categories were identified, which were grouped into each of the abovementioned theoretical categories.

**Figure 3:**
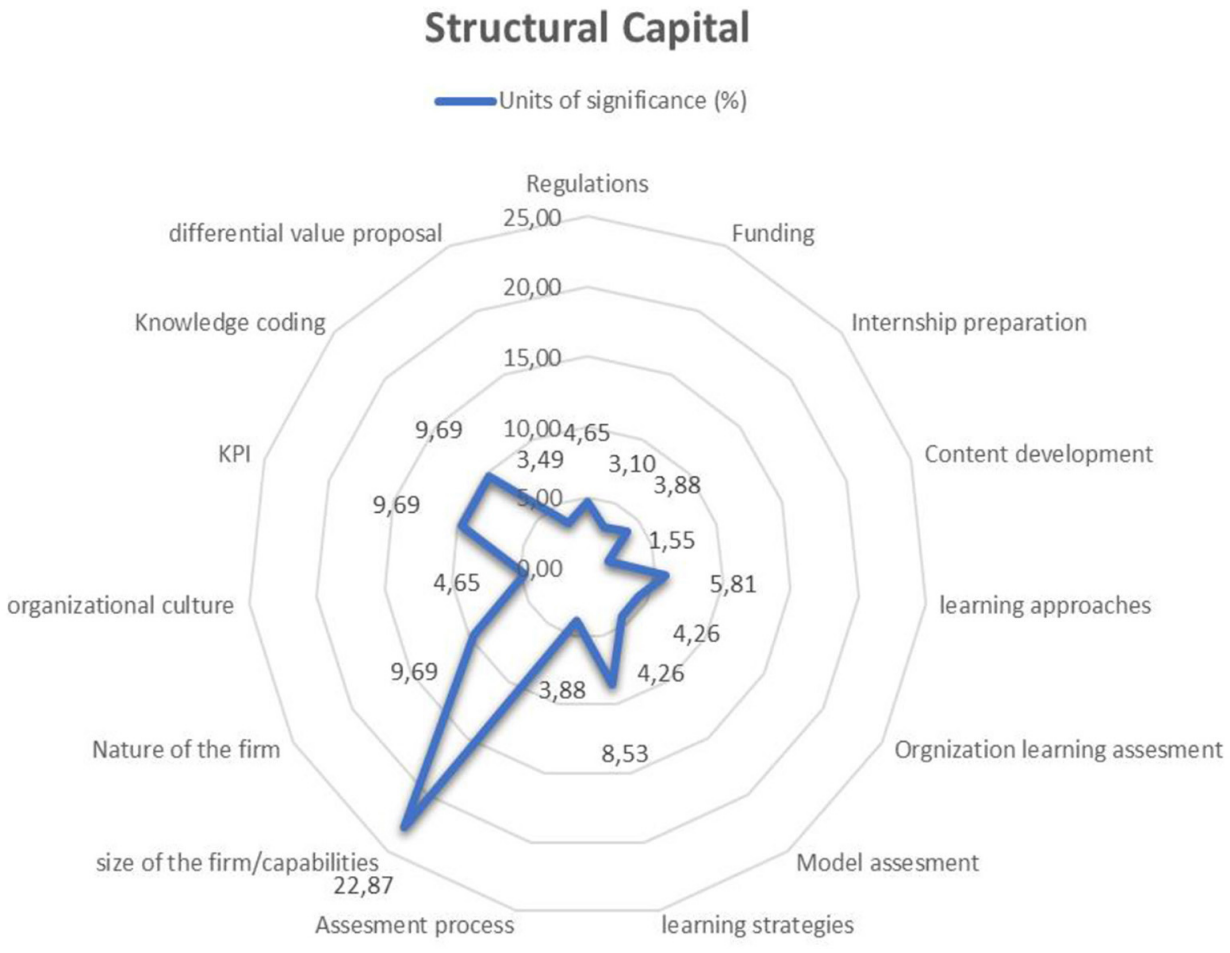
Structural capital. Source: Author's elaboration.

The structural capital built-in professional formation for the participants in the research should contain the following elements: clarity in the normativity and financing of the model, the necessary preparation process in the universities before starting the internships, and the development of contents that account for learning and approaches from the classroom and the company, having clarity in the learning evaluation models not only of the students but also of the organizations and of the model itself. This should also consider the size, capabilities, nature of the companies and their organizational culture, the measurement of the same through clarity in the KPIs, the development of knowledge codification, and the alignment with the differential value proposition that the model itself offers (Figure 3).

Some of the arguments that support these results are as follows:

“*The ease of applying what we learn depends on the size of the company and the sector in which they are, in SMEs you can bring more knowledge (Student).”*

“*The model requires harmony between the university and the companies, but as there are few companies, it is difficult for students to do their internships well applied, we have cases where they are very small companies, then they go to do one, the marketing internship, and they put them to do something about quality management or they put them to do something else, so sometimes they tend to deviate a little from the concept of the internship and they will do it more for commercial needs” (Practical Coordinator)*.

*Students can adequately apply their knowledge in a large company or a small company, or they can be in a large company and not apply any of the knowledge, the important thing is the accompaniment that we are doing (Decision makers)*.

“*There are regulatory barriers in Colombia that have very complex financial implications because in general, the dual model in all countries where it has been implemented has a strong support from the company in terms of financing the students because there is a retribution in the training process” (Program Director)*.

**Figure 4:**
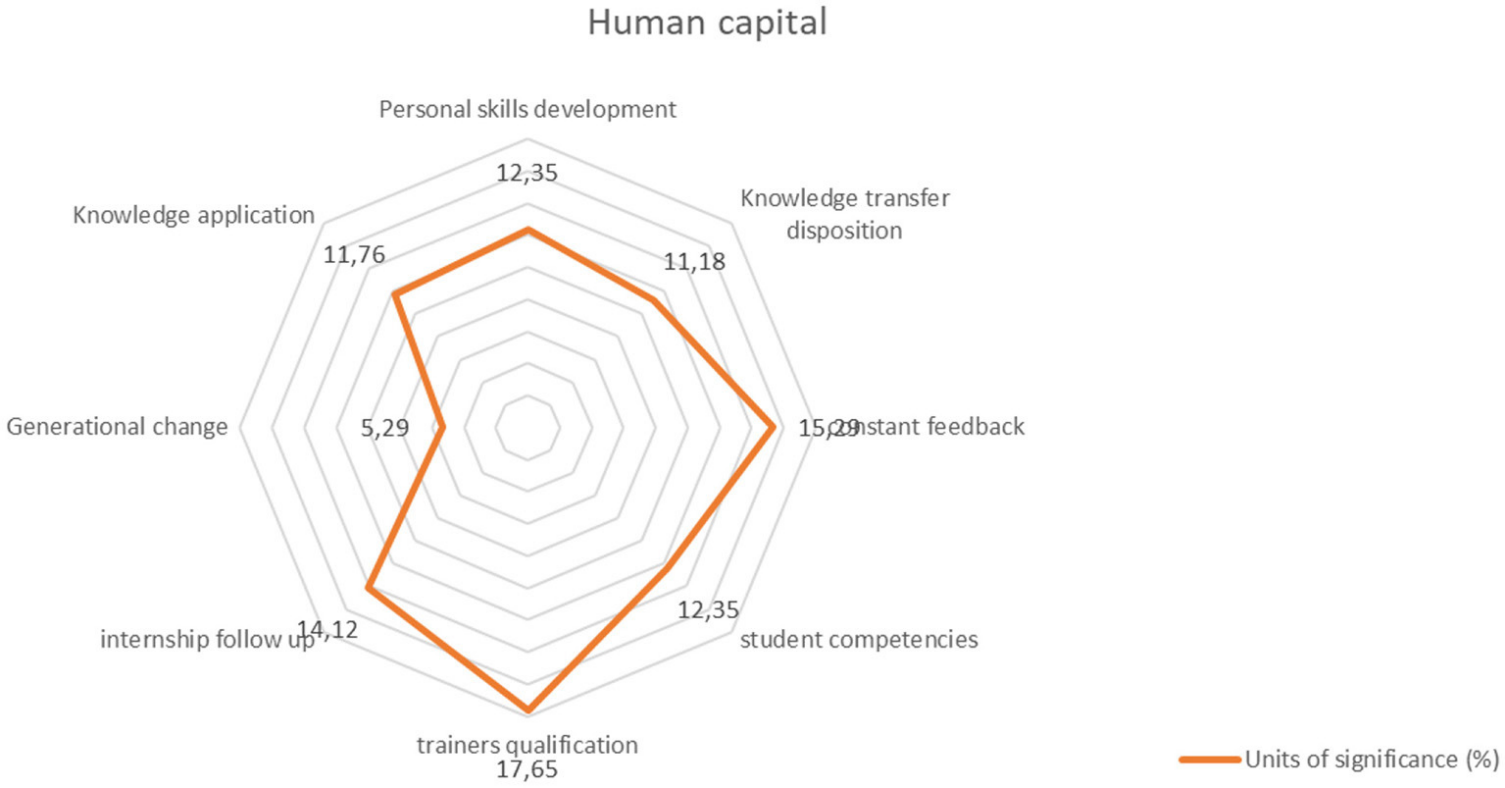
Human capital. Source: Author's elaboration.

The formation of human capital becomes the higher purpose that should mediate the academy–business relationship in professional formation, and the results show the key aspects for this to happen: the development of competencies in students, teachers, and instructors, a strong component of training in soft skills, constant feedback processes between the different actors in the process, the open willingness of employers to transfer their knowledge to students and of students to apply the knowledge acquired in the classroom, the construction of the necessary interaction to follow-up internships, and the necessary training for students to become the key candidates for generational change in organizations (Figure 4).

Some of the contributions made by participants were the following:

“*The coordination between university professors and company instructors allows students to feel that they are learning and applying their knowledge simultaneously” (managerial focus group)*.

“*It would be necessary to create a proper profile or overview of the company where the best route is outlined for students and for them to apply their work proposals” (interview* internship *coordinator)*.

“*The DHLA teacher must be an experienced researcher and use high-level bibliographic sources in his classes; (...) He must know the level of the student at each stage to identify the appropriate teaching material and use the most appropriate methods. The DHLA teacher must know in general terms the business training plans and the objectives (according to the training framework plan) of each area.” (official document DHLA_08)*

*There is a formula that is very simple and practical, and it is that in this work with the company the university must select professors to do the follow-up there, but additionally, the companies must assign mentors, these mentors work together with the professors, and they are the ones who are going to facilitate this process (Program Director)*.

**Figure 5:**
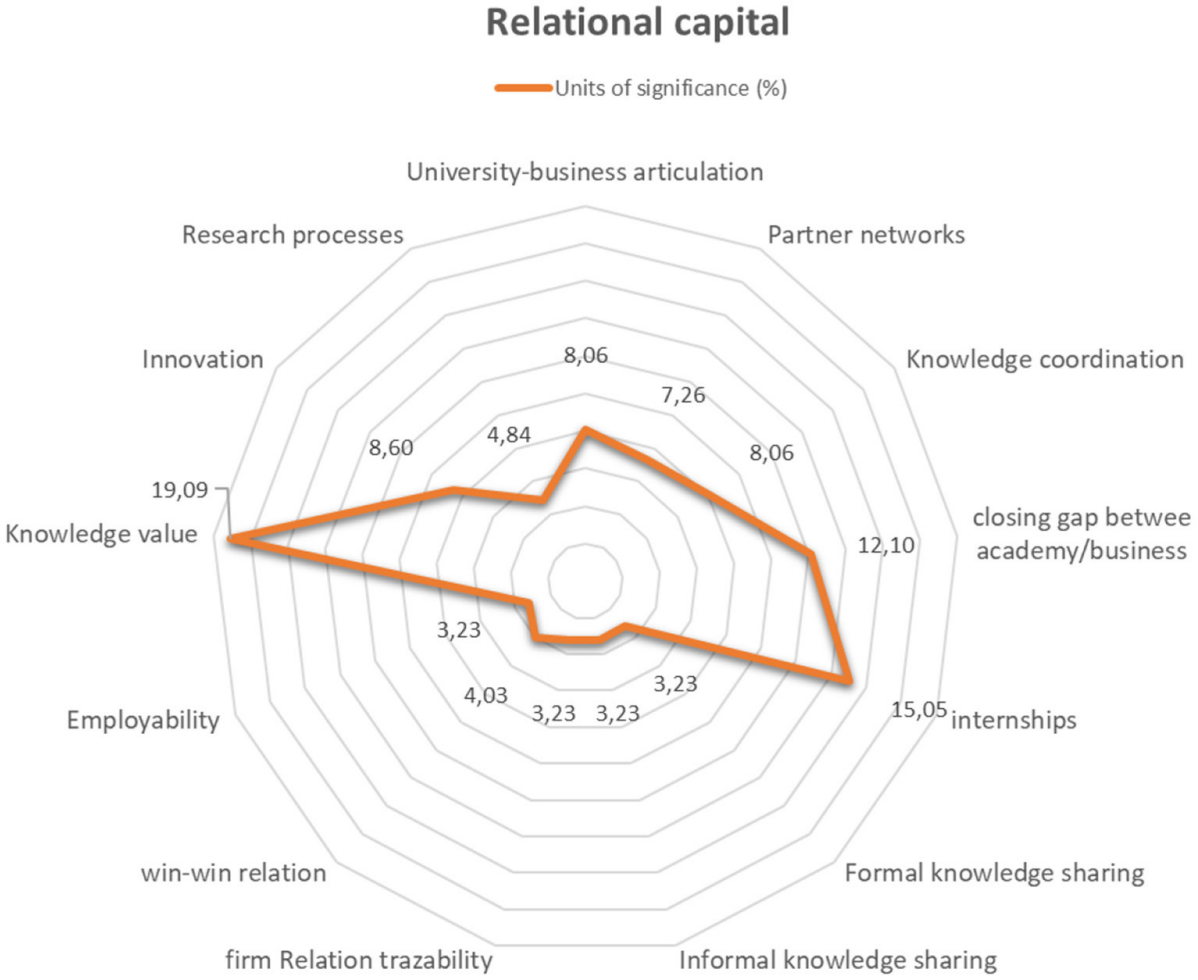
Relational capital. Source: Author's elaboration.

The construction of relational capital should be understood as the differential of this type of training compared to other existing ones. The first element is the university–business articulation, the possibility of creating networks of partners, the shared coordination of the model, the closing of the gap between academia and business in terms of time and resources, the constant development of internships for students, the development of channels for the constant exchange of formal and informal knowledge, the possibility of traceability of the training and growth of students, the bidirectional flow of knowledge, the spaces in the organizations that allow the training of students and promote employability, the recognition of the value of the knowledge generated, and the development of applied research and innovation processes (Figure 5). In this regard, the participants state that:

“*The student is implementing within the company improvements, which is a competitive advantage for the company” (entrepreneur)*.

“*Studying gives us the possibility to bring new and creative ideas to the company, helping in the creation of more value” (student)*.

“*I help them in training, but when they are already trained I want them to help me in the business as well” (entrepreneur)*.

“*With the university business model, what the company does is that it anticipates the training process of the workers and gains the person's study time with a learning process and when that person joins the company after finishing their studies they will be much more productive than another person who comes from a conventional model” (entrepreneur)*.

“*From my point of view, it depends a lot on the company where you can apply what you are seeing at the moment” (student)*.

“*The most valuable thing about the model is the opportunity to capitalize on the student's knowledge in the organizations, through the internship projects” (internship coordinator)*.

“*The company provides a contribution in knowledge management from organizational structures and the business environment to the academy in favor of competitiveness and value creation” (Program Director)*.

#### Development of the final categories—Metacategories

A total of five final categories emerged from the codification and categorization process carried out during the analysis process. The proposed categories allow comprehending of the necessary elements for the construction of intellectual capital in professional formation, understanding that this process takes place in the academy–enterprise relationship.

**Figure 6:**
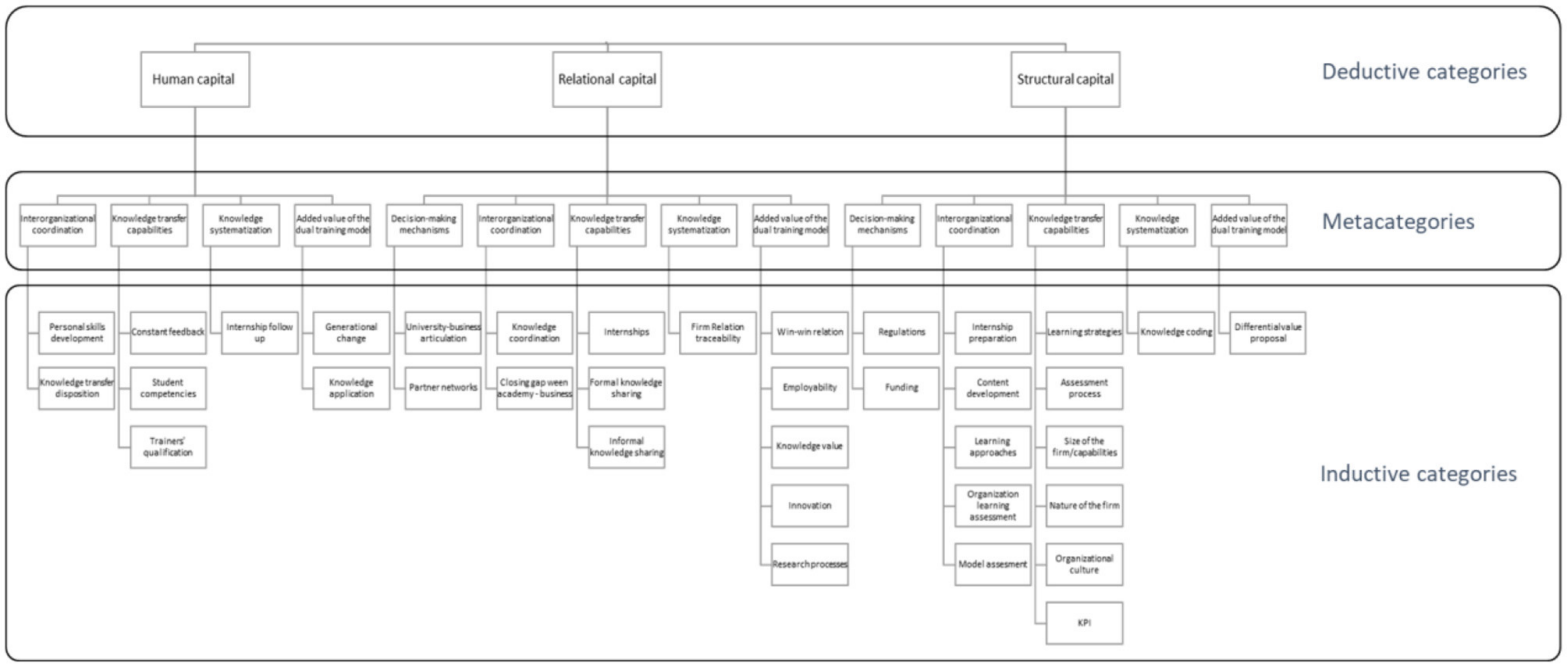
Metacategories. Source: Author's elaboration.

The categories that emerge allow explaining the necessary elements that intervene in the construction of the intellectual capital academy–enterprise relationship are as follows (Figure 6):

1. Decision-making mechanisms.
2. Interorganizational coordination.
3. Knowledge transfer capabilities.
4. Knowledge systematization.
5. Added value of the professional formation model.

## Discussion

This research has analyzed the point of view of the different actors involved in the professional formation process in Colombia, the way to build intellectual capital, from the relationship between academia and business. The results were grouped into three types of capital: relational capital, structural capital, and human capital, based on the processes of generation and transfer of knowledge for their analysis. The understanding of how intellectual capital developed from the implementation of professional formation makes it possible to explain how the gap between academia and business can be closed in a way that favors the country's economic growth.

### Decision-making mechanisms

According to the findings, the decision-making mechanisms in the professional formation processes in Colombia are linked to the structural capital necessary for its operation, and these conditions vary in different countries where the model has been implemented; in the Latin American case, specifically in Colombia, the academia–industry relations occur at the micro-level, i.e., only between institutions. Germany, on the contrary, presents different levels of relationships, being the role of the government a relevant point, stated also by Gessler (2017a) developed by Rojas Hernández et al. (2019). This becomes a challenge for Colombia, where professional formation as an educational model has been regulated for a few years and the implementation mechanisms are still poorly understood.

### Interorganizational coordination

The results show that learning spaces must be explained from their particularities and needs, it is necessary to understand the curricular model of training that must be implemented in the companies and to recognize this space as a training space rather than a practice space; on the contrary, universities must manage to incorporate the learning promoted by the business sector. Training spaces must be aligned for the proper development of training where the context plays a fundamental role (Lucas et al., 2012). Additionally, the results evidence the importance of inter-organizational dialog and the relevance of tutors, coinciding with Gessler and Hinrichs (2015).

### Knowledge transfer capabilities

Knowledge transfer processes between the classroom and business are mediated by the interest of the parties, so there must be an awareness of the generation of value from them (von Krogh et al., 2001). In this regard, the participants stated that there is a high interest in sharing knowledge by the entrepreneurs; however, this is linked to the size of the company, the spaces, and channels of dialog, and the business culture, which agrees with Rodríguez Gómez (2009) who states that knowledge transfer is strongly related to the organizational culture. Regarding the channels through which knowledge transfer takes place, the informants stated that they are mostly informal, which is supported by Eom and Lee (2010) findings, who also identified that informal channels are the most used way to transfer knowledge between the academy and business, the communication and transfer channels must be clearly defined to capture knowledge more effectively, and the motivation to interact plays an important role for the use of these channels (D'Este and Perkmann, 2011). Concerning the use of knowledge, the findings show how this is a process that is conditioned by the type of organization, its willingness to cooperate, and the structure of the educational Institution, and on this, Kanama and Nishikawa (2017) state that companies can improve their innovative performance when collaborating with academia, only if there is clarity about the value of knowledge.

Concerning the transfer, the most relevant issue is to understand what is the real value of knowledge, the results in this sense show that there is a high potential to use the knowledge generated in the academy–business relationship, and it is necessary to have all the structure for its use; in this sense, different studies raise the importance of activating this knowledge (Gupta and Govindarajan, 2000), and this requires the development of a coordinated strategy for this to occur.

### Knowledge systematization

To understand how to create intellectual capital capable of contributing to economic development from professional formation, it is necessary to implement measurement and monitoring processes that allow not only the traceability of the process but also to demonstrate the true value of training. These findings coincide with the results obtained by different authors (Hinrichs, 2014; Gessler and Hinrichs, 2015).

### The added value of the professional formation model

Professional formation value creation can be understood from different perspectives, and one of them is the possibility of generating new applied knowledge from incremental innovation processes, coinciding with the contributions of Euler (2013) regarding the implementation in other countries, and Kaiser et al. (2015), who highlights the role of networks for innovation. Thus, the academy–business relationship not only promotes cooperation and knowledge generation but also results in the generation of innovations that enable economic growth and sophistication of the productive sector (von Krogh et al., 2001; Eom and Lee, 2010). On the contrary, professional formation enables faster business response times (Gessler and Hinrichs, 2015), allows students to influence the improvement of processes through learning (Bandura, 2010), and improves knowledge transfer through collaboration (Chiaburu et al., 2010).

### Intellectual capital, professional formation, and economic growth

Based on the categories analyzed, the construction of intellectual capital through professional formation is evidenced by its human capital, structural capital, and relational capital dimensions and its impact on closing gaps between business and academia.

Human capital is built mainly from the development of personal skills and the willingness to transfer knowledge, through inter-institutional coordination, student competencies, training of instructors, and constant feedback in the category of knowledge transfer capabilities, follow-up of internships, and the generational relay and application of knowledge as evidence of the added value generated by the professional formation model.

The strengthening definition of structural capital from the dual model is demonstrated across the regulations and financing as decision-making mechanisms, the implementation of inter-organizational coordination that prepares students for the internships, develops content, formulates the learning model, and establishes both organizational and the model learning assessment. Moreover, this capital is strengthened by the ability to transfer knowledge between academia and business through the proposal and establishment of learning strategies and evaluation processes, considering the capabilities and size of companies, their nature, and organizational culture, formalized in a specific measurement system and the codification of knowledge. The added value created by the professional formation model is reflected in a differential value proposition for the parties involved.

Finally, relational capital management promotes the model strengthening through the mechanisms used for decision-making from the articulation between academia and business, and the generation of networks and their traceability. Similarly, with inter-organizational and knowledge coordination, the gap between academia and business is closed, which promotes the development of successful internships, the formal and informal knowledge exchange, and the generation of value of the professional formation is evidenced by win–win relationships, the employability of young people, the creation of innovations and knowledge, and collaborative research processes.

## Conclusion

Human capital is a determining factor in the economic growth of a country, especially in the so-called knowledge society, so it becomes a real factor of production. In this way, it is important for countries to commit in terms of investment in the education of competent human capital, research, innovation, and technological development, to improve the productive structure of the business (Hyde, 2015; Greiner et al., 2016; Stiglitz and Greenwald, 2016).

In the case of Colombia, it becomes a necessity, first, to train the existing labor force, which is low-skilled and much focused on low-value-added services, and this should be shifted into conceiving education as an endogenous factor in its economic growth formula (Thelen, 2006; Acevedo, 2007). Second, the great changes in organizational models, the speed with which enterprises grow, and the new normality resulted from the COVID-19 pandemic provide a favorable context for professional formation to become a model that accounts for these changes, especially because of the immediate response in academic training.

Consequently, a positive relationship is found between the development of intellectual capital through professional formation models and economic growth and decent work, through the closing of academia and business gaps. The comprehension of the business, economic and social needs by higher education institutions, and knowing their capacity to respond in an articulated manner with the company promote the employability of young people with better working conditions given the qualifications and experience they acquire through the model.

Similarly, the company's commitment to the promotion of professional training applied to the business context generates greater creation and strengthening of the organization's intellectual capital from its human capital dimensions, training and promoting the development of personal skills in its employees; from the structural capital by establishing mechanisms, procedures, systematization of the model, innovations, and development of the organizational culture; and from the relational capital with the articulation and relationship with the academy that allows the creation of opportunities for young people and the strengthening of the educational and business system resulting into greater economic growth and internships that promote decent work and employability of young people.

### Limitations and future research

Some limitations were found in carrying out this research that refers to the limited studies on professional training in Latin America since the studies that were found measure specific aspects of the training process and not the entire training system, creating opportunities for future research. A series of elements are key to understanding how intellectual capital generates value in organizations, turning them into more competitive companies that generate growth and development.

On the contrary, there is an opportunity to analyze the cases of other emerging economies that develop professional formation models, thus measuring the performance with the five factors identified and finally be able to determine how they have managed to reduce the academy–business gap and its impact on sustainable and economic development. Finally, it can lead as well to research in detail the vision of organizations, analyze the indicators of improvement, and monitor the results for the formation of intellectual capital over time.

## Data availability statement

The raw data supporting the conclusions of this article will be made available by the authors, without undue reservation.

## Ethics statement

Ethical review and approval was not required for the study involving human participants in accordance with the local legislation and institutional requirements. Written informed consent to participate in this study was not required from the participants in accordance with the national legislation and the institutional requirements.

## Author contributions

AV-R, AP, and MM-P: conceptualization and formal analysis. AV-R: data curation and research. AV-R and MM-P: methodology. AV-R, AP, MM-P, and DN-B: writing—original draft and writing—reviewing and editing. All authors contributed to the article and approved the submitted version.
